# In Situ Dispersion of Lignin in Polypropylene via Supercritical CO_2_ Extrusion Foaming: Effects of Lignin on Cell Nucleation and Foam Compression Properties

**DOI:** 10.3390/polym15081813

**Published:** 2023-04-07

**Authors:** Keen Hoe Ho, Xuehong Lu, Soo Khim Lau

**Affiliations:** 1 School of Materials Science and Engineering, Nanyang Technological University, 50 Nanyang Avenue, Singapore 639798, Singapore; hokh@simtech.a-star.edu.sg; 2 Singapore Institute of Manufacturing Technology, Agency for Science, Technology and Research, 5 CleanTech Loop #01-01, CleanTech Two Block B, Singapore 636732, Singapore

**Keywords:** supercritical CO_2_, foaming, polypropylene, lignin, nucleating agent

## Abstract

Supercritical CO_2_ (scCO_2_) extrusion foamed high-melt-strength (HMS) polypropylene (PP) often suffers from low cell density, large cell sizes, and poor cell structure uniformity due to the poor nucleation rates of CO_2_ in the PP. To remedy this, various inorganic fillers have been used as heterogeneous nucleation agents. Although their efficient nucleation effects have been demonstrated, the preparation of these fillers causes some adverse effects on the environment/human health or involves relatively expensive processes or non-eco-friendly chemicals. In this work, biomass-based lignin is studied as a sustainable, lightweight, and cost-effective nucleating agent. It is found that scCO_2_ could assist in situ dispersion of lignin in the PP in the foaming process, leading to significantly increased cell density, smaller cells, and improved cell uniformity. The Expansion Ratio is also simultaneously improved due to reduced diffusive gas loss. The PP/lignin foams with low lignin loadings exhibit higher compression moduli and plateau strengths than the PP foams with the same densities owing to the improved cell uniformity and probably also the reinforcing effect of the small lignin particles in cell walls. Moreover, the energy absorption capability of the PP/lignin foam with 1 wt% lignin could match the PP foam with similar compression plateau strengths; even the density of the former is 28% lower than the latter. Therefore, this work provides a promising approach to a cleaner and more sustainable production of HMS PP foams.

## 1. Introduction

Supercritical carbon dioxide (scCO_2_) as a blowing agent offers great sustainability advantages over conventional hydrocarbon blowing agents as CO_2_ is typically produced as a by-product from chemical plants, refineries, fossil fuel power plants, etc. In addition to its sustainability benefits, scCO_2_ possesses a dynamic solubility parameter, a unique characteristic of supercritical fluids, which allows for quicker diffusion and dissolution of scCO_2_ in polymer melts during scCO_2_ foaming processes [1]. Moreover, this dynamicity enables scCO_2_ to assist the dispersion of many substances in incompatible media, such as the dispersion of polar additives in non-polar polymers [2,3,4]. Polypropylene (PP) is a popular non-polar foam material for applications ranging from automotive and household products to food and protective packaging. Although scCO_2_ foaming could allow for PP foams to be fabricated more sustainably, it is plagued with various issues that cause the fabricated foams to have poor foamability and undesired foam morphology. While the use of long-chain branched high-melt-strength (HMS) PP could significantly enhance the foamability of PP [5], the undesired foam morphology caused by the low nucleation rate of CO_2_ in PP melt remains a critical issue for some continuous foaming processes, such as extrusion foaming. The resulting low cell density, large cell size, and poor cell uniformity could cause poor properties in the PP foams [6]. To address this issue, a possible approach is to utilise scCO_2_ to promote in situ dispersion of nucleating agents in PP in scCO_2_ foaming processes.

It has been widely reported that mineral fillers, such as talc [7,8], can be used as nucleating agents in scCO_2_ foaming, promoting heterogeneous nucleation by providing large surface areas to accommodate the nuclei [7]. Although these inorganic fillers are in general not costly, they may cause some environmental and health concerns. For instance, for talc, raw material extraction often involves mining activities that destroy or pollute natural habitats [9]. Furthermore, naturally occurring talc is often contaminated with asbestos, a known carcinogen, raising some concerns about the occupational health of the workers who handle these fillers [10]. Aside from these concerns, inorganic fillers also have significantly higher densities than polymers, e.g., 2.7–2.85 g/cm^3^ for talc vs. 0.88 g/cm^3^ for HMS PP. Therefore, the incorporation of these fillers in polymers may diminish the lightweight benefit of the polymer foams to some extent [11]. Recently synthetic inorganic nanoparticles, such as molecular-sieve [12] and talc nanoflakes [13,14], and lightweight synthetic polymers, such as fluoroelastomers [15] and polydimethylsiloxane (PDMS) [16], have also been demonstrated as efficient nucleating agents for scCO_2_ foaming of PP. Nevertheless, the syntheses of these nanomaterials all involve relatively expensive chemical processes or non-eco-friendly chemicals [9,17].

A potential solution to the aforementioned problems is to utilise biomass-based lignin as a sustainable, lightweight, and cost-effective nucleating agent for scCO_2_ extrusion foaming of PP. Lignin is a type of hyperbranched natural macromolecule, which is abundant, renewable, and mass-produced as a by-product from the paper pulping industry; it is extremely not costly, even more than the HMS PP that is commonly used for foaming [18]. In addition to being biodegradable and biocompatible, it is a safer and less dense (1.35–1.50 g/cm^3^ [19]) alternative to mineral additives, such as talc [20]. With adequate dispersion in PP, lignin could potentially serve as a nucleating agent due to its cavity-rich surfaces that are favourable for heterogeneous nucleation of gas cells in foaming [21], and the induced nucleation would lead to significantly higher cell density, smaller cell size, and more uniform cell size, benefiting the mechanical properties of the foams [7]. However, many reports have shown that direct mixing of commercial technical grade lignin with PP via melt compounding would result in poor dispersion of lignin in PP [22,23] owing to the polarity mismatch between the polar lignin and highly non-polar PP [24]. Since the scCO_2_ used for PP foaming could be concurrently utilised to reduce this polarity mismatch by leveraging on its dynamic solubility parameter [3], the scCO_2_-assisted in situ dispersion of lignin in PP via extrusion foaming could be a plausible solution to this problem. Herein, we demonstrate that by using a simple foam extruder attached to a mini melt homogeniser (Figure S1), the high shear force applied could work synergistically with scCO_2_ to promote in situ dispersion of lignin in PP melt. The lignin dispersion states in the foams with different contents of lignin and the morphological features of these foams are also correlated to their compression mechanical properties and energy cushioning properties. The results show that the lignin could effectively reduce diffusive gas loss and induce gas cell nucleation, and may also act as reinforcing fillers at low lignin contents. As a result, the PP/lignin foams could offer significantly more material and weight savings than PP foams without compromising their compression mechanical properties and energy cushioning properties. Therefore, the study provides a promising approach to a cleaner and more sustainable production of PP foams.

## 2. Experimental

### 2.1. Materials

The polymer used was a WB 140 grade HMS PP supplied by Borealis Gmbh. Alkaline lignin powder (product number L0082) was supplied by Tokyo Chemical Industry Co., Ltd. (Chennai, India) Both materials were used without further treatment.

### 2.2. Foam Fabrication

A designated amount of lignin (0.5, 1, 2.5, and 5 wt%, respectively) was melt-compounded with HMS PP using a Macromatex II 27-mm Co-rotating Twin Screw Extruder (Leistritz, Nuremberg, Germany). The screw speed, die temperature, and barrel temperature on the equipment were set at 135 rpm, 180 °C, and between 180–190 °C, respectively. Subsequently, the compounded material was pelletised and added into a ZSE 27 MAXX Twin Screw Foam Extruder (Leistritz, Nuremberg, Germany) that was installed with a mini melt homogeniser and a 2 mm pinhole die. With the extrusion throughput set at 7 kg/h, the CO_2_ loading and PP loading were set at 2 and 98 parts, respectively. The barrel temperature prior to CO_2_ injection was set between 170–190 °C while after CO_2_ injection, it was set at 145 °C. With the equipment settings described above, the actual polymer melt temperature at the die and the melt pressure at the flange were ~160 °C and 75 bar, respectively. At the CO_2_ injection port, the setup injected liquid CO_2_ into the barrel at ~30 bar. Due to the high-pressure environment of the foam extruder, the injected CO_2_ achieved a supercritical state in the barrel of the extruder. The diagram of the equipment setup is provided in Figure S1 of Supporting Information (SI). To fabricate PP foams with different densities as reference samples, the same processing conditions were used except that the gas loading and extruder throughput was changed to create foams with different foam densities. The variations in these parameters are shown in Table S1 in SI.

### 2.3. Characterisation

FESEM 7800F PRIME Field Emission Scanning Electron Microscope (SEM) (JEOL Ltd., Tokyo, Japan) was employed to characterise the dispersion states of lignin in PP/lignin nanocomposites before and after foaming. To avoid any morphological features from being covered by a sputtered conductive material, Low-Vacuum Secondary Electron Imaging (LVSEI) mode was used. Histograms showing lignin particle size distribution in PP/lignin foams were created by measuring the diameters (*d*) of the lignin particles in typical cross-sectional SEM images of the PP/lignin foams using ImageJ image processing software (version 1.53e, National Institutes of Health and the Laboratory for Optical and Computational Instrumentation, Bethesda, MD, USA). For each lignin loading, three 500× and three 5000× magnification SEM images were used to measure the particles with a diameter ≥1 and <1 µm, respectively. For each particle size interval in the histograms, the particle counts obtained from the three SEM images of the same magnification were summed to achieve a total count, *c*, while the foam cross-sectional areas measured from the three SEM images were also summed to achieve a total area, *α*. The particle count per volume, *P*, was then calculated using the equation $P={\left(\frac{c}{\alpha }\right)}^{3/2}$. The morphologies of the foams were examined using FESEM 7600 (JEOL Ltd., Tokyo, Japan). All the test pieces for SEM studies were prepared by cryo-fracturing liquid N_2_-chilled foam samples to expose their cross-sections. For morphological characterisation, the test pieces were further sputtered with gold at 20 mA current and 40 s exposure duration. To quantitatively characterise the foams, the extrudates were sliced along their cross-sections to obtain cylindrical samples. The expansion ratios (ER) of the foam samples were then calculated using the equation $ER=\frac{\rho_{solid}}{\rho_{foam}}$, where *ρ_solid_* and *ρ_foam_* are the density of the non-foamed and foam samples, respectively [8]. The sample densities were calculated by dividing its weight by its vernier calliper measured volume. To further observe the foam morphology and to measure the cell density, cell size, and cross-sectional area of the foams, a high-resolution 3200 dots per inch benchtop scanner was first used to optically image the cross-section of the foams. For scale reference, a vernier calliper was imaged along each sample. To distinguish the cell walls from the gas cells, the cross-section of the cell walls was stained with black ink prior to scanning. Subsequently, the measurement was conducted on the image using ImageJ image processing software. The cell size is defined as the minimum Feret diameter of the cells and the cell density was calculated using the equation $N={\left(\frac{n}{A}\right)}^{3/2}\times ER$, where *N*, *n*, *A*, and *ER* are the cell density, the number of cells in a defined region, the area of the defined region, and the Expansion Ratio, respectively [8]. To measure the compressive properties of the foam samples, Criterion Series C43 tensile tester (MTS Systems Corporation, Eden Prairie, MN, USA) with a 1 kN load cell was used and the test was conducted according to ASTM 1621-16 at 10% strain/min until 0.95 kN of force was reached. The compression plateau strength of the foams was measured from their compression curves at 20% strain. For each PP/lignin loading, the test was repeated five times and a mean from these values was reported. The energy absorption efficiency (η)-strain (ε) plots of the foams were generated from the corresponding compression curves, for which η was calculated using the equation $\eta \left(\varepsilon \right)=\frac{\int_{0}^{\varepsilon }\sigma \left(\varepsilon \right)d\varepsilon }{\sigma \left(\varepsilon \right)}$, where *σ*(ε) is the compression stress at strain ε. From the η-ε plot of a foam sample, its densification onset strain (ε_d_), i.e., the strain corresponding to peak energy absorption efficiency, was determined. The specific energy absorption of the foam was then determined as the area under the compression curve in the strain range of 0 to ε_d_. To measure the crystallinity % and melting temperature of PP and PP/lignin foams, Q10 Differential Scanning Calorimeter (DSC) (TA Instruments, New Castle, DE, USA) was used to conduct single heating and cooling cycle thermal analysis at 10 °C/min. The crystallinity % was calculated using 209 J/g as the enthalpy of melting for fully crystalline PP [25].

## 3. Results and Discussion

### 3.1. In Situ Dispersion of Lignin in PP via scCO_2_ Extrusion Foaming of PP/Lignin

**Figure 1:**
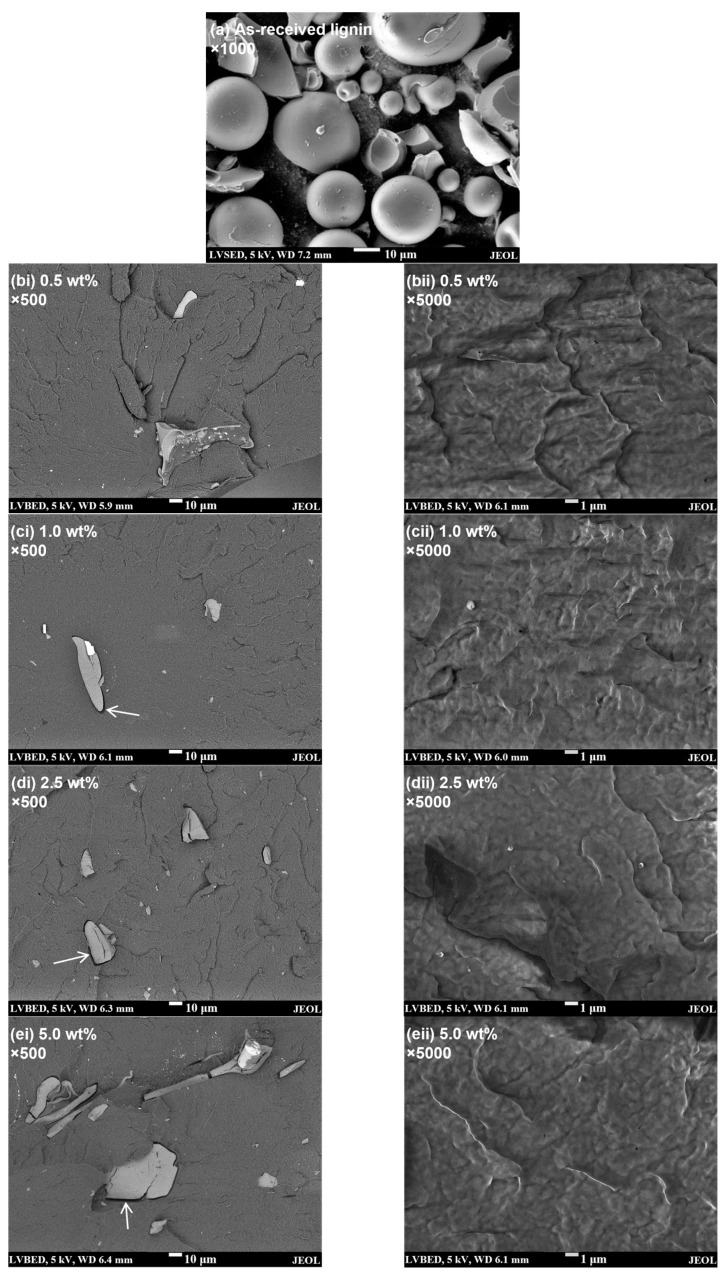
Typical SEM images showing (**a**) as-received lignin powder, and fractured cross-section surfaces of as-compounded PP/lignin composites with (**b**) 0.5, (**c**) 1.0, (**d**) 2.5, and (**e**) 5.0 wt% of lignin at (**i**) low magnification and (**ii**) high magnification.

**Figure 2:**
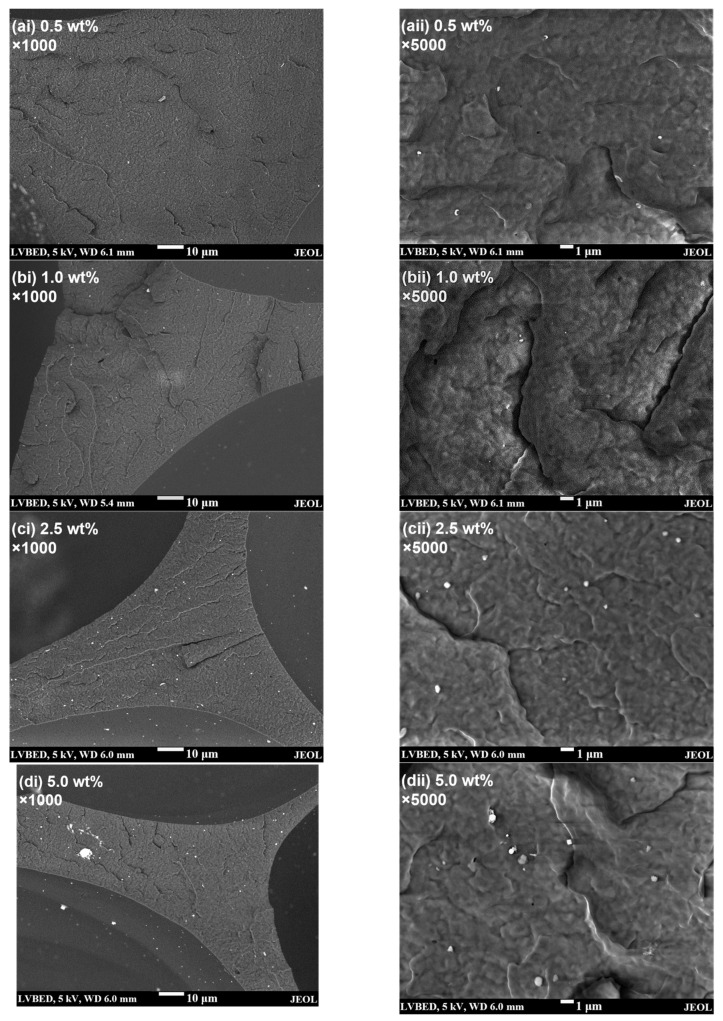
Typical SEM images showing the fractured cross-section surfaces of PP/lignin foams with (**a**) 0.5, (**b**) 1.0, (**c**) 2.5, and (**d**) 5.0 wt% of lignin at (**i**) low magnification and (**ii**) high magnification (also used for lignin particle size distribution analysis of diameter <1 µm).

In this work, commercially available lignin powder was first incorporated into PP at various loadings via melt compounding and the resultant composites were then foamed using the scCO_2_ foam extrusion process. As lignin must be adequately dispersed in PP to provide relatively large surface areas for heterogeneous nucleation, the amount of lignin incorporated into the foams is limited up to 5 wt%. Simply by melt compounding, the dispersion of the as-received lignin in PP is expected to be very poor due to their great polarity mismatch. From the SEM image in Figure 1a, it can be observed that the as-received lignin particles have an average diameter of ~35 µm. After compounding, large particles with sizes of tens of microns could still be observed in all the composites (Figure 1(bi–ei)) and very few dispersed small particles could be detected using SEM even at relatively high magnification (Figure 1(bii–eii)), indicating that the as-received lignin particles could be barely broken down into micron-sized particles merely by the high shear force of the twin screw compounder. In terms of composition dependence, the phase separation is clearly significantly more severe for the composite with 5 wt% lignin. Moreover, some of the observed large particles are seemingly made of agglomerated lignin bulks, implying poor compatibility between the lignin and PP. The effect of the polarity mismatch between lignin and PP is also evidenced by the clear interfacial gap between the lignin particle and the PP matrix (marked by the arrows in Figure 1(ci–ei)). In contrast to the very poor dispersion of lignin in the as-compounded PP/lignin composites, the SEM images in Figure 2 show that after scCO_2_ extrusion foaming, large lignin particles could hardly be observed in the PP/lignin foams; rather, few micron-sized and numerous submicron-sized particles are randomly dispersed in the PP matrix. These clearly show the capability of the scCO_2_ to compatibilise PP and lignin, which could work synergistically with the shear force provided by the twin screw to achieve significantly-improved dispersion of lignin in PP.

**Figure 3:**
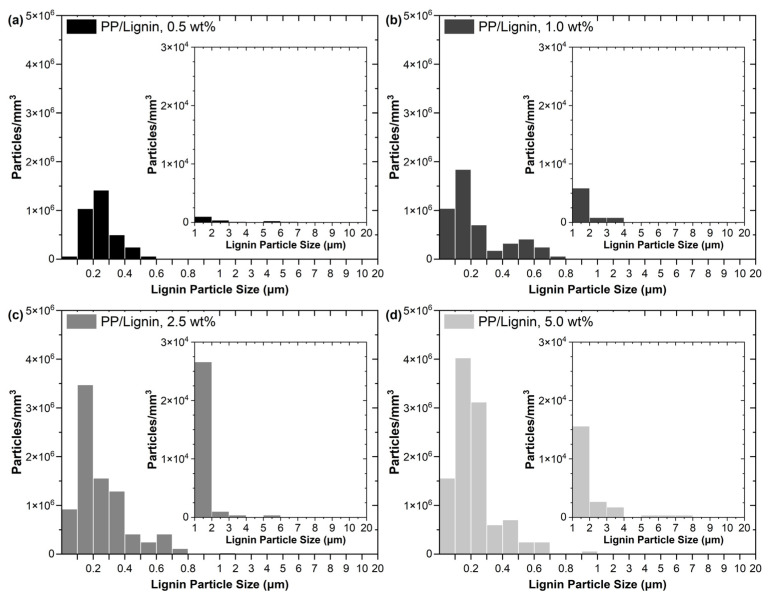
Histograms showing lignin size distributions in the PP/lignin foams with (**a**) 0.5, (**b**) 1.0, (**c**) 2.5, and (**d**) 5.0 wt% of lignin.

To further demonstrate the effectiveness in situ dispersion of lignin brought by the scCO_2_ extrusion foaming, lignin particle size distributions in the foams are presented in Figure 3 (the histogram data for each foam sample are generated from three 500× and three 5000× SEM images, cf. Figure 2(aii–dii) and Figures S2 and S3 in SI). The histograms show that the total number of micron- and submicron-sized particles per volume increases with the lignin loading, while the PP/lignin foams with low lignin contents of 0.5 and 1.0 wt% have very limited amounts of micron-sized lignin particles. The significantly-improved dispersion of lignin in PP/lignin foams would greatly increase the specific surface areas of the lignin particles, enhancing the heterogeneous nucleating effect of lignin.

### 3.2. Nucleating Effect of Lignin in scCO_2_ Foaming of PP

**Figure 4:**
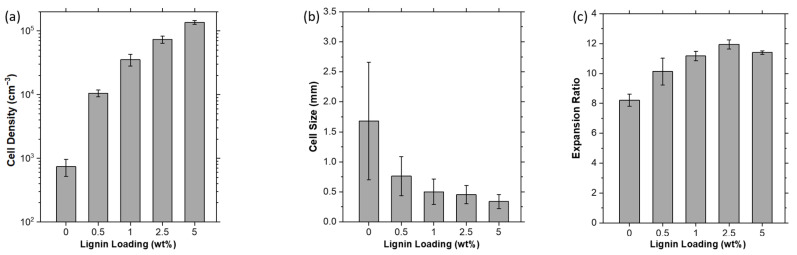
(**a**) Cell density, (**b**) cell size, and (**c**) Expansion Ratio of the PP and PP/lignin foams.

**Figure 5:**
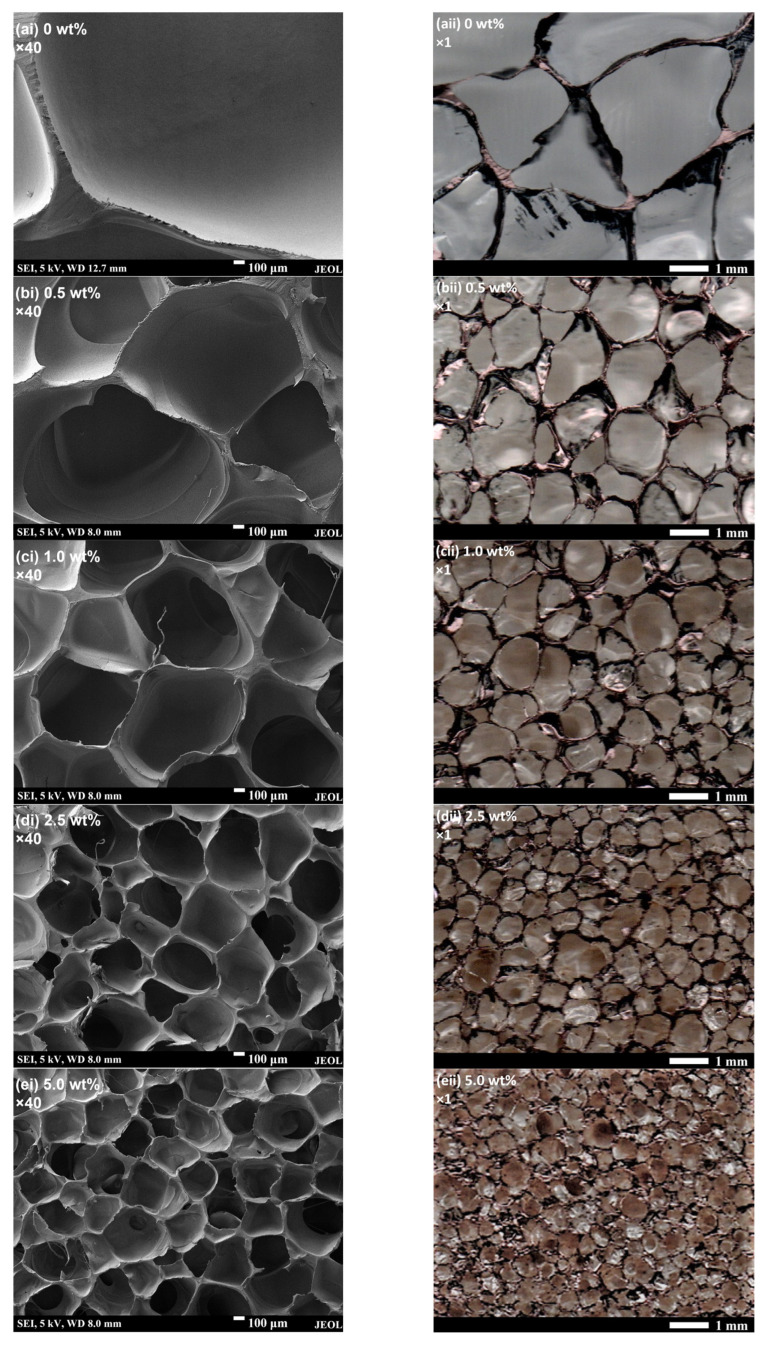
(**i**) SEM and (**ii**) optical cross-sectional images of the (**a**) PP and PP/lignin composite foams with (**b**) 0.5, (**c**) 1, (**d**) 2.5, and (**e**) 5 wt% lignin loading showing the foam morphologies.

In Figure 4, the cell density and cell size of the PP and PP/lignin foams are measured from their respective optical cross-sectional images. The representative optical cross-sectional images are as shown in Figure 5(aii–eii). Figure 4a,b shows that with a low lignin loading of only 0.5 wt%, the extruded PP/lignin foam already exhibits an order increase in cell density and >50% reduction in average cell size after the scCO_2_ foaming. As the lignin loading increases, the cell density increases and the average cell size decreases monotonically. In the tested composition range, the PP/lignin could achieve cell density improvement of up to two orders of magnitude. Moreover, the PP/lignin foams exhibit improved cell size uniformity over the PP foam. This is reflected by the error bars associated with PP/lignin in Figure 4b, which shows a generally decreasing relative standard deviation of 58.5%, 42.5%, 42.1%, 33.7%, and 34.1% as the lignin loading increases from 0 to 5 wt%. This trend signifies a reducing variability in cell size, and thereby indicates an improving uniformity in cell size as the lignin loading increases. Therefore, these results indicate that lignin could indeed serve as an effective nucleating agent. The good nucleation performance of lignin is probably due to its abundance of surface cavities and its hyperbranched structure that provides intramolecular cavities. As established in previous studies on heterogeneous nucleation theory [26,27,28], these cavities provide an additional geometry effect that further reduces the energy barrier for nucleation. Therefore, this allows gas nucleation to take place more readily, giving rise to higher nucleation rates.

In addition to the foam morphology changes, it can be observed from Figure 4c that ER also increases as the lignin content increases up to 2.5 wt% lignin loading (Figure 4c). The likely reason for this is the dispersed solid lignin particles in the molten cell walls which acted as diffusion barriers. After foaming and prior to the solidification of the cooling foam, the gas in the foam cells continues to diffuse through the cell walls and out of the foam, which typically results in diffusive gas losses and a decrease in ER. However, as the amorphous PP melt has significantly higher chain flexibility and mobility than the hyperbranched structure of lignin, the diffusion rate of CO_2_ through the solid lignin particles will be lower than in PP melt. Therefore, the lignin particles impose an obstacle in the CO_2_ diffusion path, thus lengthening the diffusion path of CO_2_ through the cell wall which causes the diffusion rate across cell walls to become lesser. Consequently, this results in a slower diffusive gas loss and thus higher ER. This increased ER with simultaneously improved cell morphology is favourable for applications where both lightweight and adequate compression mechanical properties are essential. When the lignin content is increased to 5 wt%, ER reduces slightly as the largely increased cell density results in significantly thinner cell walls that greatly shortens the diffusion path for the escape of CO_2_ gas from the cells.

The SEM cross-sectional images of the foams are also presented in Figure 5 to show the morphological features of the foams. Since the cell sizes of some foams are at the millimetre level, optical images of the foams are also presented in Figure 5. It can be seen that despite their significantly increased ER values, the PP/lignin foams still exhibit closed-cell structures although a small degree of cell coalescences could be observed at high lignin contents of 2.5 and 5 wt%. Clearly, the size and structure of the cells in the PP foam are highly irregular. In contrast, PP/lignin foams exhibit a more uniform cell size and structure. Consequently, this improved uniformity would be beneficial to the mechanical properties of the foams [29], which will be further discussed in the mechanical properties section.

### 3.3. Compression and Energy Cushioning Properties of the PP/Lignin Foams

**Figure 6:**
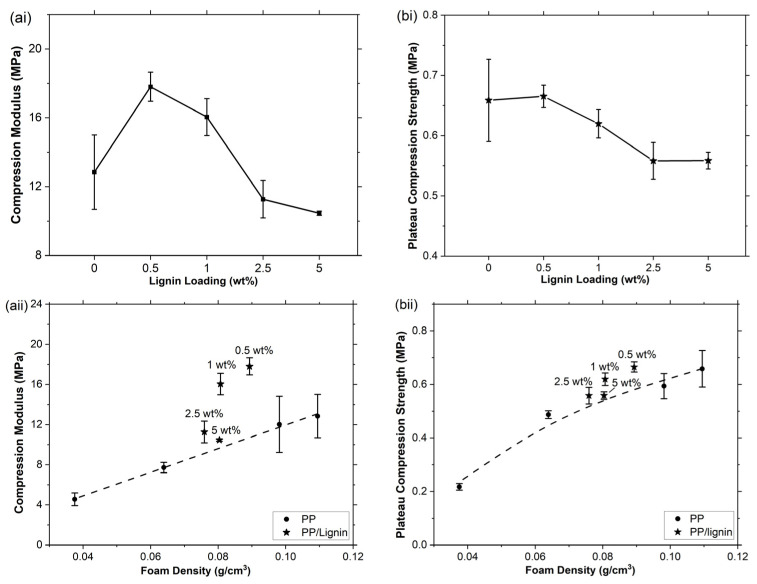
(**a**) Compression modulus, (**b**) plateau compression strength of the PP and PP/lignin foams as a function of (**i**) lignin loading and (**ii**) foam density.

Rigid polymer foams are widely used for packaging and damage-protection applications where good compression mechanical properties and large weight savings are essential. Therefore, compression modulus and plateau strength (at 20% strain) of the PP/lignin foams are measured to evaluate the mechanical performance of this type of new composite foams with PP foams as references. The results show that at lower lignin loadings of 0.5 and 1 wt%, the PP/lignin composite foams exhibit higher compression modulus than the PP foam fabricated under the same condition and that the modulus peaked at 0.5 wt% lignin loading (Figure 6(ai)), whereas the compression plateau strengths of the PP/lignin foams are lower than the PP foam except at the very low lignin loading of 0.5 wt% (Figure 6(bi)). It is worth noting that the ERs of the composite foams are all larger than the PP foam fabricated under the same condition (Figure 4c); a higher ER brings about more materials and weight savings, but it also means a higher void content that reduces the load bearing material per cross-sectional area of the foam. To exclude the effect of foam density, the compression modulus and plateau strength of a series of PP foams with different densities are plotted against their density, respectively, from which trendlines for the PP foams are established, as shown in Figure 6(aii,bii), respectively. The compression moduli and plateau compression strengths of the PP/lignin foams could then be compared with the PP foams with identical densities based on the trendlines. It is clear that with the same density, both the compression moduli and plateau compression strengths of the PP/lignin foams with low lignin loadings of 0.5 and 1.0 wt% are higher than the corresponding PP foams, and thus demonstrating the advantages of the PP/lignin foams. At higher lignin loadings, the compression moduli and plateau compression strengths of the PP/lignin foams are comparable with the corresponding PP foams.

In general, other than foam density, the compression properties of a foam could also be affected by the foam morphology, cell structure, and intrinsic mechanical properties of the cell walls [30,31,32,33]. The elastic modulus of a closed-cell macrocellular foam is mainly dependent on the bending stiffness of its cell walls. At the same foam density, the cell wall stiffness is insensitive to the average cell wall thickness, while the elastic moduli decrease with the increase in the cell size variation [34]. Therefore, with the same foam density, the enhanced compression modulus of the composite PP/lignin foams over the corresponding PP foams (Figure 6(aii)) could probably be partly attributed to the improvement in cell uniformity provided by the lignin nucleating agent. However, the cell size uniformity improves monotonically with lignin loading, whereas enhanced compression modulus is only observed at low lignin loadings. This implies that the morphological changes are unlikely to be the sole reason for the improved compression properties. As the presence of additives in semicrystalline polymers may impact the crystallisation behaviour of the polymer and lead to considerable alterations in the mechanical properties of the foams, DSC tests were performed on the PP/lignin foams to investigate how lignin affects the crystallinity % of PP during foaming. As lignin is amorphous [18], the melting peak observed in DSC is solely due to the melting of PP crystals. The information provided in Table S2 reveals that introducing lignin only results in minor reduction in PP crystallinity % at all concentrations. Furthermore, the nanocomposite’s melting temperature is not significantly affected, thus implying minimal changes in crystal size. Consequently, it can be concluded that the substantial increase in compressive strength observed in PP/lignin foams is not associated with changes in PP crystallisation behaviour. Although, lignin and PP do not form chemical bonds with each other and their interfacial interaction is limited due to polarity mismatch, the PP matrix can nonetheless still physically interact with the dispersed lignin phases. Therefore, loads on the PP matrix can still be transferred onto the rigid lignin phase albeit inefficiently due to the lack of chemical bonding and strong interfacial interaction. Furthermore, this inefficiency may be partially compensated by the scCO_2_-assisted in situ dispersion of lignin during the scCO_2_ foaming process, which increases the surface area of lignin phases and allows it to have more physical interaction with the PP matrix. Therefore, the well-dispersed small lignin particles may also act as reinforcing fillers [23] that improve the elastic moduli of the solid cell walls. This reinforcement effect may diminish at lignin loadings of 2.5 and 5.0 wt% due to the poorer lignin dispersion at higher lignin loadings (Figure 2), which results in larger lignin particles that have weaker interactions with the PP matrix owing to their smaller specific surface area.

**Figure 7:**
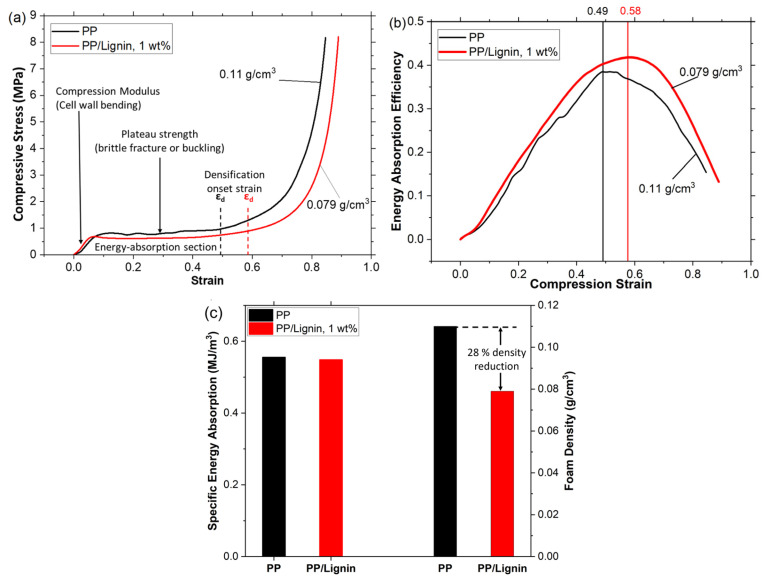
(**a**) Compression curves, (**b**) energy absorption efficiency, and (**c**) specific energy absorption (left) and density (right) of the PP and PP/lignin-1% foams.

The plateau compression strength of closed-cell foam is measured in the relatively levelled region of the compression curve (Figure 7a), in which the deformation is mainly caused by plastic buckling of the cell walls. This parameter is strongly influenced by the cell structure and the mechanical properties of the composite bulk [35]. With stiffer cell walls, more energy is required to bend the cell walls, leading to higher plateau compression strength. Additionally, a more uniform cell structure could enhance the plateau strength by allowing the load to be distributed more evenly. At low lignin loadings of 0.5 and 1.0 wt% (Figure 6(bii)), the composite foams show slightly higher plateau compression strengths than the PP foams with the same densities, which is likely to be caused by the improved cell structure uniformity and the reinforcing effects of the well dispersed submicron-sized rigid lignin particles in the cell walls. At higher lignin loadings, i.e., 2.5 and 5.0 wt%, the plateau compression strengths of the composite foams are comparable to the PP foams with identical densities, which is likely due to the interfacial debonding caused by the weak interfacial interactions between the relatively large lignin particles and PP. The debonding would create numerous voids (defects) in the cell walls, accelerating cell wall buckling. The slightly higher degree of cell coalescence at lignin loadings of 2.5 and 5.0 wt% may also contribute to a weaker cell structure that deforms more easily, causing an acceleration in cell wall buckling.

Closed-cell polymer foams are widely used as lightweight protective packaging materials as they have relatively good energy absorption capability. The energy absorption of foam could be quantified as the area under its compression curve before its densification onset strain is reached. Figure 7a shows a typical compression curve of the PP/lignin-1% composite foam in comparison with a PP foam. It can also be observed that the PP/lignin foam exhibits slightly lower compression plateau strength (at 20% strain) than the PP foam. However, in the plateau region, the compression curve of the PP foam is clearly uneven, signifying the uneven distribution of stress and foam density caused by poor cell structure uniformity. It is also important to note that the density of the PP/lignin-1% foam is 28% lower than the PP foam. From these compression curves, the energy absorption efficiencies of the foams as a function of strain could be obtained, and subsequently, the compression strain at densification onset (ε_d_) could be determined (Figure 7b) [36]. Owing to the longer and smoother plateau region of the composite foam, its energy absorption efficiency is higher than the PP foam in the tested strain range, as shown in Figure 7b. Moreover, the densification onset strain (ε_d_) of the composite foam (0.58) is also significantly higher than the PP foam (0.47), as shown in Figure 7b. This is due to the higher cell density and more uniform cell structure of the composite foam (Figure 5) that allow for a more even distribution of the compression load across the foam structure. Based on the ε_d_ obtained, the specific energy absorption of the foams could be calculated by taking the area under the compression curve before ε_d_ is reached [35]. The results are presented in Figure 7c. It can be seen that even though the density of the composite foam is 28% lower than the PP foam, they have almost the same specific energy absorption property, indicating that the PP/lignin foam would allow us to achieve a larger extent of materials and weight savings than its PP counterpart without sacrificing energy absorption capability of the foam.

## 4. Conclusions

In this work, we have successfully demonstrated that lignin is an attractive alternative to conventional nucleating agents for scCO_2_ extrusion foaming of PP. It is found that scCO_2_ could assist in situ dispersion of lignin in PP in the foaming process, enabling lignin to serve as an effective nucleating agent, i.e., giving rise to significantly increased cell density, smaller cells, and improved cell uniformity. Despite the significant increase in cell density, ER is also simultaneously improved due to the reduced diffusive gas loss caused by the presence of lignin particles in the cell walls. The PP/lignin composite foams with relatively low lignin loadings exhibit significantly higher compression moduli and slightly higher plateau strengths than the PP foams with the same foam densities owing to the improved cell uniformity and probably also to the reinforcing effect of the small lignin particles in cell walls. Moreover, due to its more uniform cell structure, the energy absorption capability of the PP/lignin foam with 1 wt% lignin could match the PP foam with a significantly higher density, allowing for substantial materials and weight savings. Considering other advantages of lignin, such as lightweight, low cost, renewable and biodegradable, and lower carbon footprint than other types of nucleating agents for polymer foaming, this work provides a promising low-cost approach to a cleaner and more sustainable production of HMS PP foams.

## Data Availability

The data presented in this study are available on request from the corresponding author. The data are not publicly available due to confidentiality policy of the institutions.

## Supplementary Materials

The following supporting information can be downloaded at: https://www.mdpi.com/article/10.3390/polym15081813/s1. Table S1: Parameters used for fabricating PP foams with different densities; Table S2: Crystallinity % and melting temperature of PP and PP/lignin foams; Figure S1: The setup of scCO_2_ foam extruder; Figure S2: Example of SEM cross-sectional low magnification images of PP/lignin composite foams with (a) 0.5, (b) 1, (c) 2.5, and (d) 5 wt% of lignin used for lignin particle size distribution analysis of diameter ≥1 µm; Figure S3: Further examples of SEM cross-sectional images of PP/lignin composite foam at (a) 0.5, (b) 1, (c) 2.5, and (d) 5 wt% used for lignin particle size distribution analysis of diameter (i) ≥1 µm and (ii) <1 µm.
