# Supplementary material for: In Situ Dispersion of Lignin in Polypropylene via Supercritical CO2 Extrusion Foaming: Effects of Lignin on Cell Nucleation and Foam Compression Properties
Source: Polymers (Basel). 2023 Apr 7;15(8):1813. doi: 10.3390/polym15081813 (PMC10145137; doi:10.3390/polym15081813)
Supplement: Supplementary file 1 [file polymers-15-01813-s001.zip › polymers-2278918-supplementary.pdf]

Table S1. Parameters used for fabricating PP foams with different densities.

**PP foam density, (g/cm^3)**	0.109	0.098	0.064	0.038
**CO_2 loading (%)**	2	2	2.5	4
**Extruder throughput (kg/h)**	7	7	5	7.5

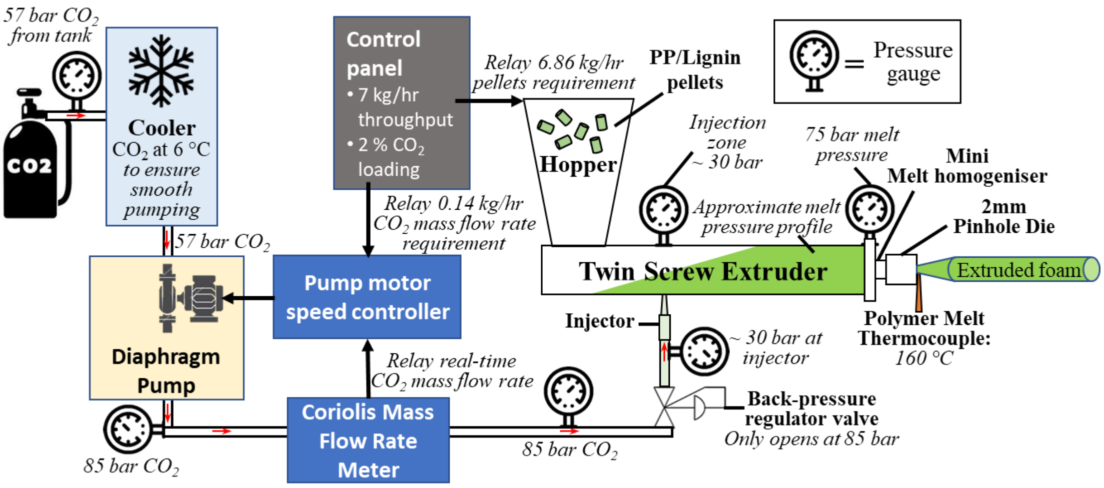

Figure S1. The setup of scCO_2 foam extruder.

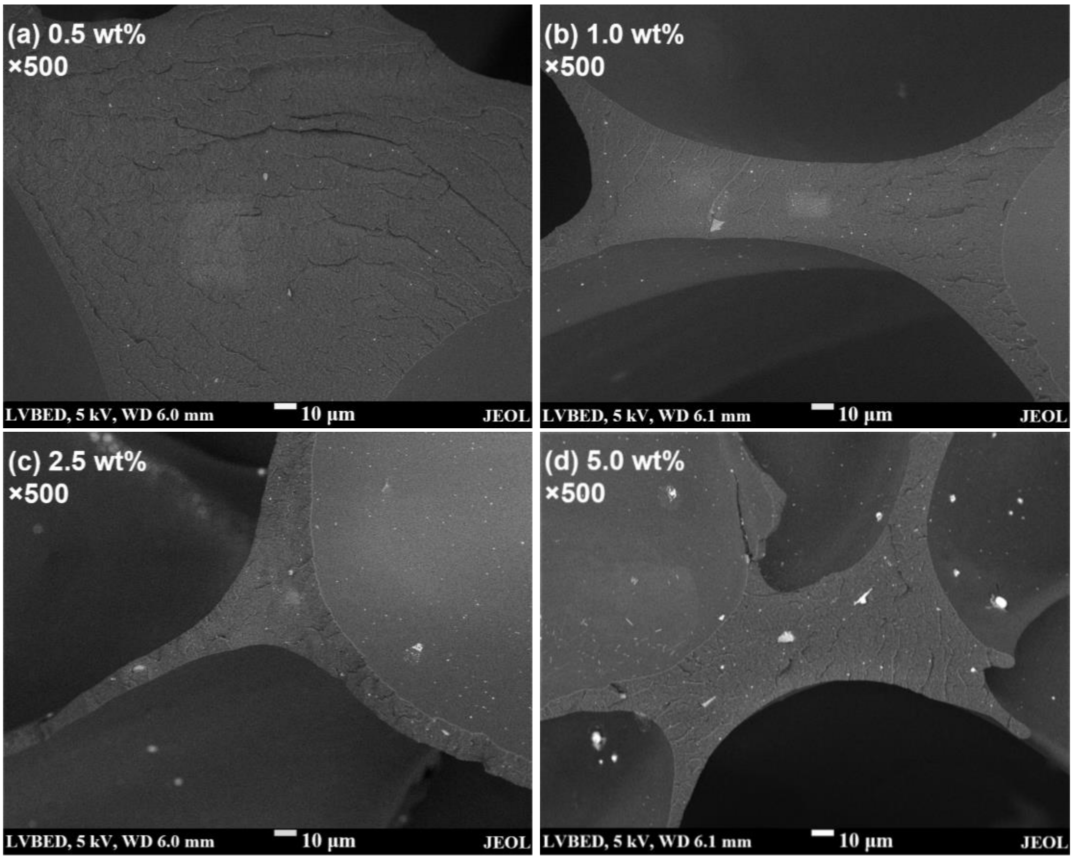

Figure S2. Example of SEM cross-sectional low magnification images of PP/lignin composite foams with (**a**) 0.5, (**b**) 1, (**c**) 2.5, and (**d**) 5 wt% of lignin used for lignin particle size distribution analysis of diameter ≥ 1 µm.

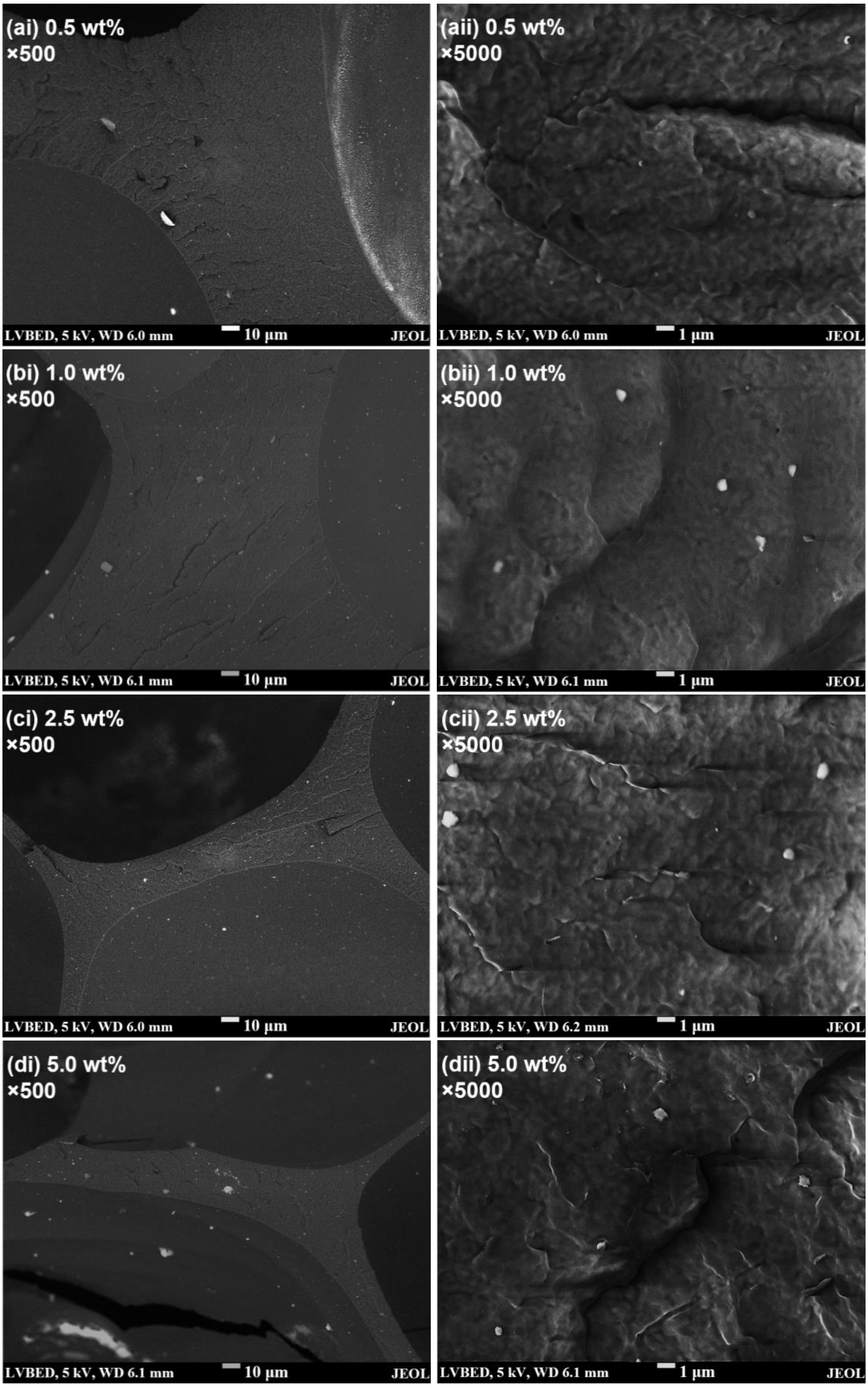

Figure S3. Further examples of SEM cross-sectional images of PP/lignin composite foam at (**a**) 0.5, (**b**) 1, (**c**) 2.5, and (**d**) 5 wt% used for lignin particle size distribution analysis of diameter (**i**) ≥ 1 μm and (**ii**) < 1 μm.

Table S2. Crystallinity % and melting temperature of PP and PP/lignin foams.

**Lignin loading (%)**	0.0	0.5	1.0	2.5	5.0
**Crystallinity (%)**	39.8	40.1	38.8	40.4	39.6
**Melting Temperature (°C)**	159.1	159.0	159.1	158.7	158.5
